# Analysis of Quality of Clinical Practice Guidelines for Otorhinolaryngology in China

**DOI:** 10.1371/journal.pone.0053566

**Published:** 2013-01-18

**Authors:** Zhe-wen Zhang, Xiao-wen Liu, Bai-cheng Xu, Su-yang Wang, Li Li, Ying-ying Kang, Yu-fen Guo

**Affiliations:** 1 School of Basic Medical Sciences, Lanzhou University, Lanzhou, China; 2 Department of Otolaryngology-Head and Neck Surgery, The Second Hospital of Lanzhou University, Lanzhou, China; 3 Ministry of Health, Gansu province, Lanzhou, China; 4 Department of Otolaryngology-Head and Neck Surgery, The Gan Su Provincial Hospital, China; University of Montreal, Canada

## Abstract

**Objective:**

To evaluate the quality of clinical practice guidelines (CPGs) for otorhinolaryngology in China.

**Materials and Methods:**

A systematic search of relevant literature databases (CBM, WANFANG, VIP, CNKI, China Guideline Clearinghouse) published between 1978 and March 2012 was undertaken to identify and select CPGs related to otorhinolaryngology. Four independent reviewers assessed the eligible guidelines using the Appraisal of Guidelines for Research and Evaluation (AGREE II) instrument. Their degree of agreement was evaluated using the intraclass correlation coefficient (ICC).

**Result:**

From 170 citations, 21 relevant guidelines were included. The overall agreement among reviewers was moderate (ICC = 0.87; 95% confidence interval [CI], 0.78–0.91). The scores for each of the AGREE domains were the following: “scope and purpose” (mean ± standard error [SE] = 45.4±4.4; ICC = 0.92), “stakeholder involvement” (mean ± SE = 30.4±3.1; ICC = 0.81), “rigor of development” (mean ± SE = 20.9±2.8; ICC = 0.87), “clarity of presentation” (mean ± SE = 48.8±3.7; ICC = 0.80), “applicability” (mean ± SE = 12.6±1.7; ICC = 0.72), and “editorial independence” (mean ± SE = 6.2±0.8; ICC = 0.76). Three guidelines (14%) mentioned updates, and the average update frequency was 7 years. None used the GRADE system.

**Conclusion:**

The quality of otorhinolaryngology guidelines in China is low. Greater efforts are needed to provide high-quality guidelines that serve as a useful and reliable tool for clinical decision-making in this field.

## Introduction

Clinical practice guidelines (CPGs) are defined as “statements that include recommendations intended to optimize patient care that is informed by a systematic review of evidence and an assessment of the benefits and harms of alternative care options.” [1] The intention of CPGs is to provide clinicians with explicit recommendations on how to manage health conditions and reduce the use of unnecessary, ineffective, or harmful interventions [2]. Approximately 2500 guidelines are estimated to be in existence [3]. However, the potential of these CPGs to improve patient care and resource use mainly depends on their quality and the rigor of the guideline development process. In fact, several recent studies found that the methodological quality of clinical guidelines was highly variable. The problem is that there are many poor-quality CPGs that are not based on the best evidence [4], [5], [6]. Therefore, assessing the quality of CPGs using formal methods is necessary.

The Appraisal of Guidelines for Research and Evaluation (AGREE) instrument is an appraisal tool and validated instrument that has been endorsed by leading producers, raters, and compilers of international CPGs [7], [8]. The purpose of the AGREE instrument is to provide a framework for assessing the quality of CPGs. In 2011, Zhang et al. used AGREE to assess the quality of pediatric CPGs in China, and the quality scored moderate in that study [9]. Chen et al. reviewed 269 CPGs published in China, but the quality scored low [10]. The quality of Chinese CPGs for otorhinolaryngology is unknown. The aim of the present study was to systematically review the quality of CPGs related to otorhinolaryngology published in the peer-reviewed Chinese medical literature and provide recommendations for guideline development in this field.

## Methods

### Identification of guidelines

We performed a computerized search of four major academic databases (Chinese Biomedical Literature database (CBM), WANFANG database (Chinese Medicine Premier), VIP information (Chinese Scientific Journals database), China National Knowledge Infrastructure (CNKI)) and the China Guideline Clearinghouse in China for CPGs for otorhinolaryngology published from 1978 to March 2012. We used *guideline* as a subject heading in the CBM abstract database and three terms, *guideline* or *specification* or *consensus*, in the title of the papers in the other three full-text databases. The references retrieved included guidelines that met the definition of a guideline proposed by the Institute of Medicine [1]. We excluded Chinese versions of foreign CPGs and adapted versions of CPGs from other countries.

### Appraisal instrument

AGREE II, an update of the original AGREE instrument, is widely used to assess CPGs across various medical specialties [2], [11]. It contains 23 key items organized into six methodological domains: “scope and purpose” (items 1–3), “stakeholder involvement” (items 4–7), “rigor of development” (items 8–14), “clarity of presentation” (items 15–18), “applicability” (items 19–21), and “editorial independence” (items 22–23). Each item in a domain is scored from 1 (strongly disagree) to 7 (strongly agree). The score for each domain is obtained by summing all of the scores of the individual items in a domain and then standardizing them as the following: *(obtained score - minimal possible score)/(maximal possible score - minimal possible score)*. The maximal possible score for each domain is the number of questions multiplied by the number of reviewers multiplied by 7 (i.e., the score for “strongly agree”). The minimal possible score for each domain is the number of questions multiplied by the number of reviewers multiplied by 1 (i.e., the score for “strongly disagree”).

Four independent reviewers who did not participate in the development of the guidelines were trained in CPG appraisal using the AGREE instrument. Two of them are clinicians in otorhinolaryngology and the other two are graduates students. Each reviewer independently reviewed the quality of each CPG using the AGREE instrument. Whenever disagreement arose, a third observer, designated by consensus by the working group, issued the final verdict.

### Statistical analysis

We performed a descriptive statistics analysis using the calculation of the total score by each reviewer and the score per domain. The data obtained by assessing the guidelines after applying the AGREE II instrument and intraclass correlation coefficients (ICCs) were considered to assess inter-rater reliability within each domain [12]. The independent-sample test was used for comparison of two samples and one-way Anova was used for multi-comparison. Statistical significance was considered with *p*<0.05. The software used for the analysis was SPSS 11.0.

## Results

### Guideline characteristics

**Figure 1:**
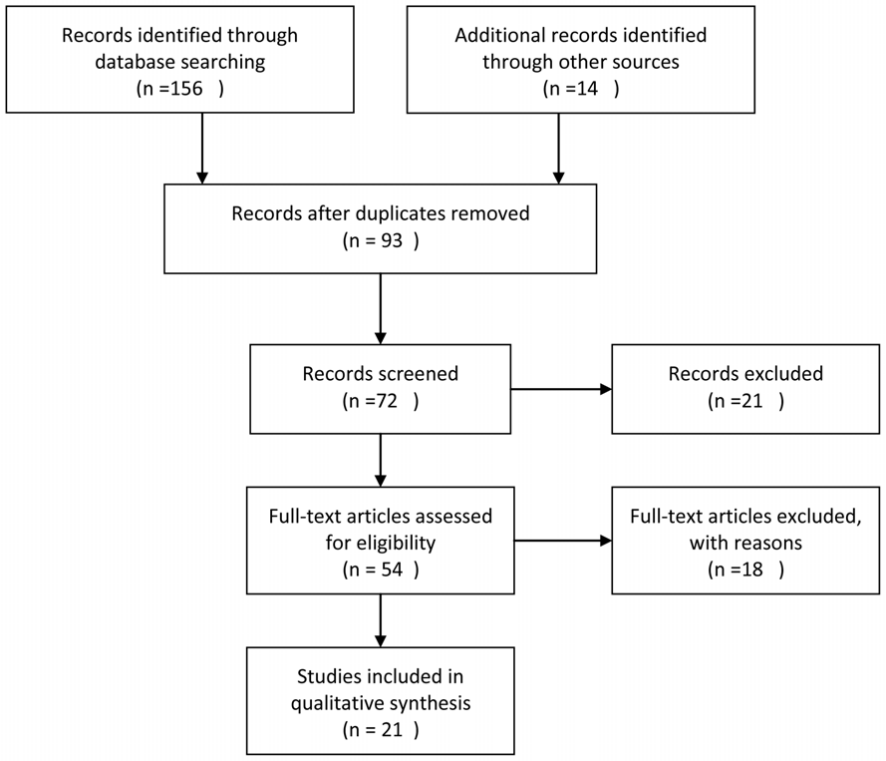
Flowchart of systematic search.

The initial search identified 170 documents. After applying the inclusion and exclusion criteria, 21 guidelines were eventually considered for inclusion (Fig. 1). The demographic characteristics for each of the included guidelines are presented in Table 1. Fifteen (71%) guidelines were developed by medical societies, followed by hospitals (24%) and governmental agencies (5%). Eleven (52.3%) guidelines were developed by two professional organizations or agencies. Twelve (57%) guidelines never reported the number of authors, and three (14.3%) guidelines did not have more than 10 authors. Fourteen (66.7%) guidelines cited references (range, 3–41; mean, 13). Six (28.6%) guidelines provided the name of the contact person, and only five (23.8%) guidelines provided contact emails. Two (9.5%) guidelines attached additional documents. Three (14%) guidelines mentioned the time and frequency of updates. The average total number of pages of the guidelines was 3.6 (range, 1–8).

**Table 1:** Clinical practice guidelines for otorhinolaryngology.

Title	Year of publication	Number of organizations	Update/period	Developed methods	Number of references	Topics covered
Guidelines on tinnitus diagnosis and treatment	2009	6	Not mentioned	Literature review and consensus	3	Diagnosis/Treatment
Guidelines for the diagnosis and treatment of pediatric otitis media	2008	2	Not mentioned	Literature review	10	Diagnosis/Treatment
Clinical practice guidelines for management of pediatric otitis media	2007	1	Not mentioned	Not mentioned	Not mentioned	Diagnosis/Treatment
Guidelines on sudden sensorineural hearing loss diagnosis and treatment	2005	2	1 time/8 years	Literature review	Not mentioned	Diagnosis/Treatment
Clinical practice guidelines for the management of infant hearing screening	2009	2	Not mentioned	Literature review and consensus	41	Screening
Clinical practice guidelines on cochlear implants	2007	1	Not mentioned	Consensus	Not mentioned	Technology
Guidelines on cochlear implants	2003	2	Not mentioned	Literature review and consensus	6	Diagnosis/Treatment
Consensus guidelines for cochlear implants	2003	3	Not mentioned	Consensus	Not mentioned	Diagnosis/Treatment
Guidelines on chronic nasal sinusitis diagnosis and treatment	2008	2	Not mentioned	Literature review and consensus	8	Diagnosis/Treatment
Guidelines for pediatric sinusitis	2005	1	Not mentioned	Literature review	13	Diagnosis/Treatment
Guidelines for pediatric allergic rhinitis	2011	3	Not mentioned	Literature review	5	Diagnosis/Treatment
Guidelines for allergic rhinitis	2009	2	3 times/18 years	Literature review and consensus	4	Diagnosis/Treatment
Guidelines for allergic rhinitis	2004	2	Not mentioned	Not mentioned	Not mentioned	Diagnosis/Treatment
Consensus guidelines for allergic rhinitis	2010	2	Not mentioned	Consensus	4	Diagnosis/Treatment
Consensus guidelines for specific immunotherapy of allergic rhinitis	2011	2	Not mentioned	Consensus	28	Diagnosis/Treatment
Guidelines on the treatment of nasal endoscopy	2008	1	Not mentioned	Literature review	10	Treatment
Guidelines for diagnosis and treatment of nasal cavity and sinus diseases in aircrew and student pilots	2011	1	Not mentioned	Literature review	29	Diagnosis/Treatment
Guidelines on nasopharyngeal carcinoma	2007	1	Not mentioned	Literature review and consensus	Not mentioned	Diagnosis/Treatment
Guidelines for pediatric obstructive sleep apnea-hypopnea syndrome	2007	2	Not mentioned	Literature review and consensus	7	Diagnosis/Treatment
Guidelines for adult obstructive sleep apnea-hypopnea syndrome	2012	1	1 time/9 years	Literature review and consensus	18	Diagnosis/Treatment
Guidelines for obstructive sleep apnea-hypopnea syndrome and uvulopalatopharyngoplasty	2002	2	Not mentioned	Not mentioned	Not mentioned	Diagnosis/Treatment

### Appraisal of guidelines

A total of 21 Chinese otorhinolaryngology CPGs were evaluated using the AGREE II, with four reviewers per guideline. Table 2 shows the results for each of the guideline areas after being evaluated using the AGREE II instrument. The overall agreement among reviewers for the evaluation with the AGREE II instrument was moderate (ICC = 0.87; 95% confidence interval [CI] = 0.78–0.91). Below we describe the appraisal results according to the AGREE II domains.

**Table 2:** Guideline score (Mean±SE) and Guideline distribution (%) according to score on each of the domains assessed by the AGREE II instrument.

Domain	Mean±SE	Categories of quartiles of the highest score possible			
		<25%	25–50%	50–75%	>75%
Scope & Purpose	45.4±4.4	19.1	42.9	38.1	0
Stakeholders	30.4±3.1	42.9	47.6	4.7	4.7
Rigour	20.9±2.8	74.4	23.8	4.7	0
Clarity	48.8±3.7	4.7	74.4	14.3	9.5
Applicability	12.6±1.7	90.5	9.5	0	0
Editorial independence	6.2±0.8	100	0	0	0

**Scope and purpose:** The score for this domain reflects the degree to which the overall objectives of the guidelines, clinical questions covered, and patients to whom the guidelines were meant to apply were specifically described [13]. Most of the guidelines performed relatively well in this domain. The ICCs showed high agreement between appraisers (0.92). The mean score for this domain was 45.4%, and four of the guidelines (19%) scored below 25%.

**Stakeholder involvement:** This domain evaluates the degree to which CPGs represent the views of their intended users. It evaluates whether individuals from all relevant professional groups are represented, whether the views and preferences of the target population (e.g., patients, public, etc.) have been sought, and whether the target users of the guidelines were well-defined [13]. The mean score was 30.4%, with only two of the CPGs (9.5%) scoring over 50%, suggesting the poor involvement of stakeholders in guideline development. None of the guidelines involved patients in the development process or was piloted among end-users. The ICCs were moderate (0.81).

**Rigor of development:** This domain is considered the most important. It evaluates the integrity of the development process, including the reporting of the search methodology, evidence selection criteria, methods used to formulate recommendations, risk and benefit assessment, and links between evidence and recommendations, external review, and updating mechanisms [13]. The mean score for this domain was only 20.9%. Fifteen guidelines (74.4%) scored below 25%. The ICCs were moderate (0.87). Over 80% of the guidelines did not mention any database in their search strategy and did not include a system to evaluate the quality of the evidence or grade the strength of the recommendations. Three guidelines (14.3%) mentioned updates, but none described a procedure for updating the guidelines. Among these three guidelines, the average update frequency was 7 years. Five guidelines (23.8%) were externally reviewed prior to publication.

**Clarity of presentation:** This domain assesses the clarity of the guidelines and whether the recommendations are specific and unambiguous, whether different management options are clearly presented, whether key recommendations are easily identifiable, and whether the guidelines are supported by tools for their application [13]. Overall, the mean score for this domain was the highest (48.8%). Only one of the guidelines (4.7%) scored <25% for this domain. The ICCs were moderate (0.80).

**Applicability:** This domain evaluates issues that are pertinent to guideline implementation. More specifically, it considers whether the guidelines describe facilitators and barriers to their application, whether the potential resource implications of applying the recommendations have been considered, and whether the guidelines present monitoring or auditing criteria [13]. The mean score for this domain was 12.6%, and 19 of the guidelines (90.5%) scored below 25%. None of the guidelines discussed cost implications. The ICCs were moderate (0.72).

**Editorial independence:** This domain addresses conflicts of interest, specifically whether the guidelines were editorially independent from the funding body and whether potential conflicts of interest were reported for the members of the guideline development group [13]. The score on this domain was the lowest (6.2%). All guidelines were scored below 25%. Twenty (95.2%) guidelines did not report whether they received funding or not. The only guidelines that received funding from governments failed to report whether or not the views of the funding body influenced the content of the guidelines. The ICCs were moderate (0.76).

### Stratification of CPG quality

Table 3 presents the means of the domain quality scores from focus of the guideline (guidelines targeting ear, nose or others), year of publication (≥2008, <2008), type of development group (government, medical society or hospitals), reporting of update (mentioned, not mentioned) and object (children, adult). There was a slight difference in scope and purpose domain quality related to focus of guideline or object of guideline. The scores from guidelines available in recent five years were significantly higher than those from the other group on five domains with three domains, scope and purpose, stakeholder and applicability. The score from CPGs developed by government groups was lowest compared with the other two groups on five domains. No significant difference was obtained whether or not guidelines were updated.

**Table 3:** Mean (±SE) AGREE scores by subgroups.

Subgroups	Domain (Mean±SE)					
	Scope & Purpose	Stakeholders	Rigour	Clarity	Applicability	Editorial
Parts						
Ear(n = 8)	40.4±7.5	27.4±4.2	17.1±4.6	44.6±7.6	13.9±3.9	7±1.5
Nose(n = 9)	54.1±4.4	30±2.5	20.9±1.5	47.7±2.3	12.2±1.1	6±0.8
Others(n = 4)	36±13.5	37.5±14.0	28.3±11.7	59.8±11.5	10.8±3.8	6.3±3.2
P values	*p* = 0.001	*p* = 0.263	*p* = 0.124	*p* = 0.181	*p* = 0.449	*p* = 0.339
Year of publication						
2002–07(n = 10)	38.7±6.8	27.5±3.4	19.3±3.7	48.2±5.4	10.9±2.9	6.9±1.6
2008–12(n = 11)	51.5±5.2	33.1±5.1	22.3±4.4	49.4±5.4	14.1±1.7	5.1±0.5
P values	*p* = 0.001	*p* = 0.009	*p* = 0.109	*p* = 0.617	*p* = 0.006	*p* = 0.052
Type of development group						
Government (n = 1)⋇	42	28	9	43	5	5
Medical society (n = 15)	43.1±5.6	32.1±4.3	21±3.4	47.7±4.2	12.3±1.6	5.9±0.9
Hospitals (n = 5)	53.2±6.9	26±1.3	22.8±6.1	53.4±9.7	15±5.1	7.2±2.2
P values	*p* = 0.004	*p* = 0.001	*p* = 0.412	*p* = 0.264	*p* = 0.305	*p* = 0.261
Reporting of update						
Mentioned (n = 3)	42±18.5	41.7±18.7	27.7±17.9	50.7±21.2	13±4.4	5.3±1.3
Not mentioned (n = 19)	46±4.3	28.6±2.2	19.7±1.9	48.5±3.0	12.5±1.8	6.3±0.9
P values	*p* = 0.745	*p* = 0.349	*p* = 0.520	*p* = 0.874	*p* = 0.863	*p* = 0.105
Object						
Children (n = 8)	49.4±6.8	28.5±4.1	20.4±2.8	46.5±3.2	13.1±2.3	5.1±0.5
Adult (n = 9)	41.4±7.5	32.3±6.4	19.2±5.8	45.7±6.9	9.9±2.0	5.3±1
P values	*p* = 0.037	*p* = 0.172	*p* = 0.603	*p* = 0.761	*p* = 0.108	*p* = 0.617

^⋇^n = 1, the group was not chosen for comparison.

## Discussion

**Figure 2:**
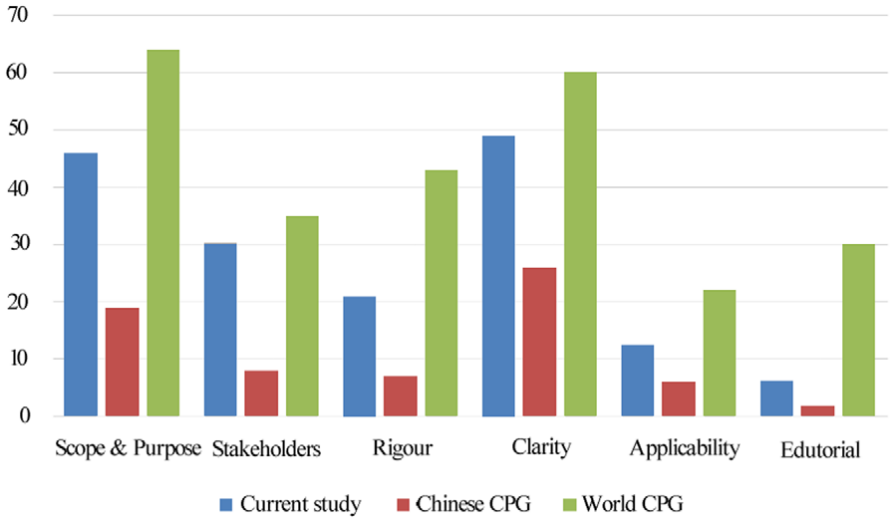
Comparison of the quality of the guidelines studied in this report with those studied by Chen (Chinese CPG) and Alonso-Coello (world CPG). This graph depicts AGREE scores: (A) Mean domain scores as reported in this study. (B) Mean domain scores evaluated on clinical guidelines in China by ChenYL. (C) Mean domain scores as reported by Pablo Alonso-Coello.

This is the first systematic evaluation of the quality of published guidelines related to otorhinolaryngology in China using a standardized appraisal instrument. Overall, the guidelines for this field had poor quality. Fig. 2 presents a comparison, by AGREE domain, of the guidelines assessed by Chen [10] and Alonso-Coello et al [14]. Alonso-Coello et al. reported the quality of CPGs across a wide range of healthcare topics published since 1980. Chen assessed the quality of Chinese CPGs published in the peer-reviewed medical literature. All of the domain scores in our study were lower than the world average, particularly in the “rigor of development” and “editorial independence” domains, and higher than Chen. Low-quality guidelines have low scores, particularly within the domains of “rigor of development,” “applicability,” and “editorial independence.” Specifically, a low score for “rigor of development” is worrisome because this domain may be a stronger indicator of quality than any of the other domains of the instrument. Clinical practice guidelines are used as a tool for clinicians and patients to make clinical decisions, so using a standardized appraisal instrument to assess the quality of CPGs is very important.

The present study showed that the quality domains with acceptable scores were “scope and purpose” and “clarity of presentation.” Most of the guidelines described their specific and focused clinical questions and target populations well. Little improvement in the “scope and purpose” domain was found over time in the most recent 5 year period (Table 3). This can be improved by providing specific information and clear summaries.

The “rigor of development” domain had a low score. First, most of the documents did not report the systematic methods of searching for and selecting evidence or did not describe specific evaluation or recommendation systems. Second, few of the guidelines were externally reviewed prior to publication. Third, none of the guidelines provided evidence of pilot testing, which is an important issue that would ensure that the guideline can be clinical utilized. Fourth, only 14.3% of the guidelines mentioned updates, and the average update frequency was 7 years. Furthermore, none of the guidelines described procedures for updating the guidelines. Guidelines become obsolete within a mean period of 3.6 years, and the validity of all guidelines should be reevaluated every 3 years [15]. Therefore, no significant difference was obtained whether or not guidelines were updated in the present study (Table 3).

Similarly, the scores in the “stakeholder involvement” domain were low. These low scores reflect the lack of multidisciplinary teams. Half of the guidelines were developed by only two professional organizations or agencies, and less than 1% of the guidelines had methodologists. Moreover, none involved patients in the development process or was piloted among end-users. Burgers et al. reported that the quality of guidelines developed by government-funded organizations was higher than those developed by professional and specialist societies [16]. However, CPGs developed by government groups were scored as lowest on five domains in stratification of guidelines (Table 3).This outcome might be explained by poor reporting and a lack of transparency about the number of development groups.

The lowest scores on “applicability” and “editorial independence” were particularly conspicuous. These findings appear to be fairly widespread among the CPGs. The low scores may be the result of poor reporting and could be avoided if the guideline authors provided a book section or support that addressed the process concerns listed in the documents. In China, conflict of interest statements are a widely accepted policy in medical journals, but such statements are still not widely used in CPG development. The developers of CPGs need to pay more attention to these domains during the development process.

Much time and effort have been spent developing evidence-based guidelines to improve clinical practice [17], [18], [19], [20]. Unfortunately, in our present review, none of the guidelines in this field can be considered evidence-based. Moreover, none of the guidelines used the GRADE system. The need to improve the quality of evidence and strength of recommendations for the implementation of CPGs should be emphasized [17]. Efforts should be made to train guideline producers in methodology to enhance the average quality of CPGs for otorhinolaryngology in China. The cooperation of professional associations and groups within institutions dedicated to quality of care, such as the Chinese GRADE center that was established by the GRADE Working Group in 2011 at Lanzhou University, should be sought [21]. Government agencies and general and subspecialty medical societies in China have paid increasingly more attention to the role and significance of guidelines in healthcare, and the developers of guidelines should adhere more closely to the AGREE instrument when developing or updating their guidelines.

### Study Limitations

First, most Chinese guidelines have been historically derived from consensus conferences or expert opinions. Such documents are potentially useful in practice in China, especially for their emphasis on key clinical management questions. We recognize that this was a selective search for all potential guidelines in this field, so we might have included some poor-quality guidelines. Second, we may have missed guidelines published in book, booklet, or government document form, which may understate the quality of Chinese CPGs. Third, the AGREE II instrument only assesses the reporting of the different items and not the content validity of the recommendations. Lastly, another potential limitation is that the guideline developer could include some of the items listed in AGREE in process, but didn't report it.

### Conclusions

The quality of Chinese otorhinolaryngology guidelines published from January 1, 2002, to March 1, 2012, is low but seems comparable to the world average. The AGREE II instrument is feasible for appraising available guidelines. The AGREE II instrument should be adopted by China as the preferred method of guideline process assessment.

## Supporting Information

Checklist S1 **PRISMA 2009 Checklist.doc.**
(DOC)
